# How successful are mutants in multiplayer games with fluctuating environments? Sojourn times, fixation and optimal switching

**DOI:** 10.1098/rsos.172176

**Published:** 2018-03-21

**Authors:** Joseph W. Baron, Tobias Galla

**Affiliations:** Department of Theoretical Physics, School of Physics and Astronomy, University of Manchester, Manchester M13 9PL, UK

**Keywords:** evolutionary games, sojourn times, fixation dynamics, switching environments

## Abstract

Using a stochastic model, we investigate the probability of fixation, and the average time taken to achieve fixation, of a mutant in a population of wild-types. We do this in a context where the environment in which the competition takes place is subject to stochastic change. Our model takes into account interactions which can involve multiple participants. That is, the participants take part in multiplayer games. We find that under certain circumstances, there are environmental switching dynamics which minimize the time that it takes for the mutants to fixate. To analyse the dynamics more closely, we develop a method by which to calculate the sojourn times for general birth–death processes in fluctuating environments.

## 1. Introduction

Models of evolutionary dynamics frequently involve randomness, and the timing of birth, death and mutation events is statistical. The modelling framework most commonly used in evolutionary dynamics, game theory, epidemiology and population dynamics is that of a Markovian birth–death process (e.g. [1,2]). These processes capture the so-called intrinsic stochasticity (or demographic noise) in finite populations. More traditional approaches (e.g. [3,4]), based on deterministic rate equations, neglect all randomness and are valid formally only in the limit of infinite populations. Deterministic models are analysed relatively easily using tools from nonlinear dynamics; however, they do not capture fluctuation-driven phenomena such as fixation and extinction. These features can be characterized only within a stochastic model, and the analysis is frequently based on methods from non-equilibrium statistical mechanics [5,6].

Often the relative growth rates or interaction coefficients between the different types of individuals themselves fluctuate in time, as environmental conditions change. More precisely, by environmental conditions we mean the playing field upon which the individuals compete. This playing field determines the relative fitnesses of the individuals. Fluctuating environments lead to a second layer of stochasticity, in addition to the intrinsic stochasticity in finite populations. Examples of fluctuating environments include variation of external conditions (e.g. temperature, pH, presence or absence of nutrients), or targeted intervention, such as phases of antibiotic treatment (e.g. [7–12]). Biological examples include the dynamics of persister cells in bacterial populations [9,13], and the emergence of phenotype switching [14,15]. Theoretical work exists to study such systems [7,14,16,17], but often disregards intrinsic stochasticity or focuses on growth in fluctuating environments. We are mostly interested in the dynamics of fixation in fluctuating environments, which is not accounted for in the deterministic approach. We model the environmental states as being discrete, but one could also model such systems with continuous environmental noise. We note the works [18–20], based on large-deviation theory and the so-called Wentzel-Kramers-Brillouin method to compute first-passage times in systems with continuously varying environments.

Previous work [21] addressed the case of two-player evolutionary dynamics in switching environments with discrete states, and an analytical framework was constructed to calculate fixation probabilities and times. In the context of evolutionary games, a switch in the state of the environment is characterized by a sudden change in the entries of the pay-off matrix. For example, in one environment one may have a coexistence game, and in the other a coordination game. The analysis focused on games with linear pay-offs and two-player interaction. Even for this relatively simple case, unexpected behaviour was observed owing to the environmental switching; for example, fixation probabilities of an invading mutant may be maximized for intermediate switching rates of the environmental process.

The purpose of this work is to study fixation of mutants in evolutionary multiplayer games in fluctuating environments. In multiplayer games, each individual interacts with more than one other individual in each instance of the game, and the resulting pay-off or fitness functions become nonlinear in the composition of the population. This can generate multiple non-trivial fixed points of the resulting replicator equations [22]; these are the equilibria of selection at which different species coexist and have the same fitness. These equilibrium points shape the dynamics and outcome of evolution in the presence of demographic noise. We are interested in the interplay between selection, random drift and environmental fluctuations in this nonlinear scenario. We approach this in a general setting and in the context of a stylized minimal model. While we do not aim to model an immediate application, we believe our approach is the most suitable to identify the main phenomena and principal mechanisms. However, it is clear that the scenario of multiplayer interaction is of importance for a number of applications, and potentially more prevalent in the real world than two-player games. The latter are often analysed as an approximation as they are mathematically easier to handle. Applications of multiplayer games have been surveyed extensively in, for example, [23,24]. These include modelling in ecology (e.g. biological markets and auctions [25–27]), population genetics (e.g. dynamics of the Medea allele, and ‘playing the field’ in the context of the sex-ratio game [28,29]), and in the social sciences (e.g. public good games [30,31]).

Multiplayer games in finite populations have of course been investigated in the literature (e.g. [32,33]). While most existing studies focus on one fixed multiplayer game, we study cases in which the game is shaped by an external fluctuating environment. Specifically, we look at situations in which there are two environmental states. This can be thought of as modelling the presence or absence of some external stress. Under certain circumstances, we observe that the time that it takes for a mutant to take over a population of wild-types (i.e. to reach fixation) can be lower in the case of switching environments than in either fixed environment. To do this, we develop a theory for the analytical calculation of sojourn times for arbitrary two-species birth–death processes in randomly switching environments. We then apply it to understand fixation dynamics in fluctuating three-player games, and study the success of an invading mutant in an existing population of wild-types.

The remainder of this paper is organized as follows. In §2, we define the components of the model, in particular the birth–death dynamics, the environmental process and the set-up of a multiplayer game. We introduce sojourn times for general birth–death processes in switching environments in §3; these can be calculated analytically and full mathematical details can be found in the electronic supplementary material. We then study fixation phenomena in three-player games in §4, before we present our conclusions and an outlook in §5. Further technical details of the model set-up and the analysis, as well as results from some further test cases, can be found in the electronic supplementary material.

## 2. Model definition

### 2.1. Evolutionary dynamics and environmental switching

As in [21] we consider a population of *N* individuals. The size of the population does not vary in time. Each individual can be of one of two types, *A* or *B*; we will occasionally refer to these as ‘species’. The population is assumed to be well mixed, i.e. every individual in the population can interact with any other individual. The state of the population is therefore fully specified by the number of individuals of type *A*, which we write as *i*∈{0,…,*N*}. The number of individuals of type *B* is *N*−*i*. Evolution occurs through a discrete-time birth–death process, governed by transition probabilities $\omega_{i}^{\pm }$ from state *i* to states *i*±1, respectively. More precisely, $\omega_{i}^{+}$ is the probability to find the population in state *i*+1 in the next time step if it is currently in state *i*. A similar definition applies for $\omega_{i}^{-}$, and $1-\omega_{i}^{+}-\omega_{i}^{-}$ is the probability for the population to remain in *i*.

To model environmental influence, we assume that the rates of the birth–death process within the population depend on a discrete state *σ* of the environment.

We write $\omega_{i,\sigma }^{\pm }$ for the probability to transition from *i* to *i*±1 if the environment is in state *σ*. Crucially, we assume that the process of the environmental variable is Markovian and that its transition rates do not depend on the state, *i*, of the population. We write *μ*_*σ*→*σ*′_ for the probability that the state of the environment changes to *σ*′ in the next time step, if it is currently in state *σ*. We stipulate that the switching probability of the environment is independent of the state of the population *i*. This would seem appropriate for the types of systems described in the Introduction. Finally, we assume that the states of both the population and the environment can change in any one time step (i.e. the two processes are not mutually exclusive).

In our model, the dynamics of the population occurs via a standard Moran process [1,2,34]:

2.1 $$\begin{array}{rl} \omega_{i,\sigma }^{+} & =\frac{i(N-i)}{N^{2}}\frac{f_{A}^{\sigma }(i)}{f^{\sigma }(i)}\\ \;\text{and}\;\omega_{i,\sigma }^{-} & =\frac{i(N-i)}{N^{2}}\frac{f_{B}^{\sigma }(i)}{f^{\sigma }(i)}, \end{array}\}$$

where $f_{A}^{\sigma }(i)$ denotes the reproductive fitness of species *A* in a population with *i* individuals of type *A* and when the environment is in state *σ*. The quantity

2.2 $$f^{\sigma }(i)=\frac{if_{A}^{\sigma }(i)+(N-i)f_{B}^{\sigma }(i)}{N}$$

is the mean fitness in the population if the environment is in state *σ*. The states *i*=0 and *i*=*N* are absorbing states of the population dynamics for all environmental states, i.e. $\omega_{i=0,\sigma }^{+}=\omega_{i=N,\sigma }^{-}=0$. The above birth and death rates depend on the state of the environment, *σ*, through the fitnesses $f_{A}^{\sigma }(i)$ and $f_{B}^{\sigma }(i)$. These in turn are derived from pay-offs in a multiplayer game via the common exponential mapping [1] $f_{A}^{\sigma }(i)=e^{\beta \pi_{A}^{\sigma }(i)},\;f_{B}^{\sigma }(i)=e^{\beta \pi_{B}^{\sigma }(i)}$, where *β*≥0 denotes the intensity of selection, and where $\pi_{A}^{\sigma }(i)$ and $\pi_{B}^{\sigma }(i)$ are the pay-offs to individuals of either type in the multiplayer game in environment *σ*. The formalism as introduced unto here is identical to that of [21]. This earlier work focuses on the two-player case. Here, we explore the new behaviour seen in multiplayer games.

### 2.2. Multiplayer games

We assume that individual-based interactions take place between *n* players, where *n* is a fixed integer, 2≤*n*≤*N*. We will write *a*_*j*,*σ*_ for the pay-off to an individual of type *A* if they face *j* other individuals of type *A* and *n*−1−*j* individuals of type *B* in such an encounter of *n* players. The pay-off to a player of type *B* is *b*_*j*,*σ*_ if they play against *j* individuals of type *A*, and *n*−1−*j* (other) players of type *B*. These pay-offs depend on the environmental state *σ*.

This information is summarized in the following pay-off matrix (cf. [22]):

2.3 $$\begin{array}{cccccc} & A\cdots A & A\cdots AB & \cdots & AB\cdots B & B\cdots B\\ A & a_{n-1,\sigma } & a_{n-2,\sigma } & \cdots & a_{1,\sigma } & a_{0,\sigma }\\ B & b_{n-1,\sigma } & b_{n-2,\sigma } & \cdots & b_{1,\sigma } & b_{0,\sigma } \end{array},$$

and we have $\pi_{A}^{\sigma }(i)=\sum_{j=0}^{n-1}a_{j,\sigma }H^{A}(i,j)$, and $\pi_{B}^{\sigma }(i)=\sum_{j=0}^{n-1}a_{j,\sigma }H^{B}(i,j)$. The quantity *H*^*A*^(*i*,*j*) is the probability that precisely *j* individuals among the *n*−1 opponents of a player of type *A* are also of type *A*, in a population with *i* type *A* individuals in total. Similarly, *H*^*B*^(*i*,*j*) is the probability for an individual of type *B* to face precisely *j* individuals of type *A*, and *n*−1−*j* players of type *B*. Detailed expressions can, for example, be found in [22].

To analyse the evolutionary dynamics of games in finite populations, it is often useful to first consider the limit of an infinite population, although stochastic effects are suppressed in this limit. Writing *x*=〈*i*〉/*N*, where angular brackets denote an ensemble average, and keeping the environment fixed for the time-being, one finds the following dynamics in the limit N→∞:

2.4 $$\dot{x}=\omega_{\sigma }^{+}(x)-\omega_{\sigma }^{-}(x),$$

where $\omega_{\sigma }^{\pm }(x)$ is obtained from $\omega_{i,\sigma }^{\pm }$ by obvious substitutions. Equation (2.4) can formally be derived from the leading order of an expansion in the inverse system size, or equivalently from the Kramers–Moyal expansion of the relevant master equation; see [5,6] for details. We will mostly be interested in the fixed points of equation (2.4). The rates $\omega_{\sigma }^{\pm }(x)$ contain a factor of *x*(1−*x*). This is because evolutionary events affecting the state of the population require two individuals of the different types (one selected for reproduction, the other for removal). As a consequence, one always has the trivial fixed points at *x*=0 and *x*=1. Further fixed points can exist at locations *x*^⋆^ (0<*x*^⋆^<1) for which $\pi_{A}^{\sigma }(x^{⋆})=\pi_{B}^{\sigma }(x^{⋆})$. The underpinning deterministic flows of multiplayer games can then, qualitatively, be classified according to the number and stability of these internal fixed points.

Below we will focus on three-player games with a designated deterministic structure. We choose the values of the pay-off matrix elements, *a*_*j*,*σ*_ and *b*_*j*,*σ*_, so as to have fixed points in specific locations; a prescription for doing this is given for general *n*-player games in the electronic supplementary material.

**Figure 1.**
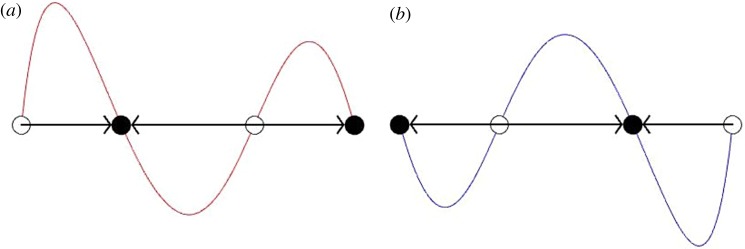
Deterministic flow in the two environments, we plot $\dot{x}$ as a function of *x*; see text for details. The internal fixed points are located at *x*^⋆^=0.3 and *x*^⋆^=0.7. The environment *σ*=1 is shown in (*a*) and *σ*=−1 is shown in (*b*). The fixed points have opposite stability in the two environments. There are only two options for choosing the direction of flow in the two environments, given that the fixed points remain in the same locations: the direction of flow is the same in both environments or the direction of flow is opposite. The most interesting is the former but we also discuss the latter in the electronic supplementary material. We also discuss the method of choosing pay-off matrix elements such that fixed points are at designated locations in the electronic supplementary material.

A typical example of the resulting deterministic flow can be found in figure 1, where we plot $\dot{x}$ as a function of *x*. We show a case of a game with (non-trivial) fixed points at *x*^⋆^=0.3 and *x*^⋆^=0.7, but in which the deterministic flow has opposite directions in the two environments. In figure 1 and in all subsequent figures below, we have always set *b*_*j*,*σ*_=1 (*j*=0,1,2) in both environments (*σ*=±1). Effectively, this is an overall normalization of pay-off, individuals of the wild-type *B* always have *π*_*B*_≡1, irrespective of the composition of the population. The remaining pay-off matrix elements, *a*_*j*,*σ*_ are then calculated as described in the electronic supplementary material. We use *β*=1/2 in all sections except §4.4.

While our theoretical approach applies to an arbitrary number of environmental states, we primarily focus on systems with two environmental states. This can be thought of as being due to the on–off nature of some external influence (e.g. the presence or the absence of an antibiotic in a sample of bacteria, or another binary stress). The most natural choice from the modelling point of view is then a set of two environments with fixed locations of possible equilibrium points and with opposite directions of flow in the two environments, turning stable fixed points into unstable ones and vice versa. Generalization to more environmental states is possible, but there is then no natural choice for the direction of the flow. In the main paper, we focus mainly on three-player games with two internal fixed points. Additional scenarios are discussed in the electronic supplementary material, including *n* player games with *n*>3 and cases in which the direction of flow is the same in both environmental states.

## 3. Fixation probabilities, fixation times and sojourn times

### 3.1. Fixation probability and fixation times

We will now turn to the dynamics in finite populations, and consider situations in which one single mutant is placed in a population of *N*−1 wild-type individuals, i.e. the initial condition is *i*=1. While the deterministic flow of the dynamics in the limit of infinite populations may have internal fixed points, the outcome of evolution in a finite population will necessarily be extinction or fixation of the invading mutant; through genetic drift the population will eventually reach one of the absorbing states, *i*=0 (extinction of the mutant) or *i*=*N* (fixation), and no further dynamics occurs. To analyse these processes, we study the probability that the mutants fixate (as opposed to going extinct), and the mean time that they take to do so.

The fixation probabilities and fixation times in switching environments have already been calculated in [21], and we briefly summarize the results here. Writing *φ*_*i*,*σ*_ for the probability that the system reaches fixation (*i*=*N*), given that it currently contains *i* individuals of type *A* and is in environment *σ*, one has

3.1 $$\varphi_{i,\sigma }=\sum_{\sigma^{\prime }}\mu_{\sigma \to \sigma^{\prime }}[\omega_{i,\sigma^{\prime }}^{+}\varphi_{i+1,\sigma^{\prime }}+\omega_{i,\sigma^{\prime }}^{-}\varphi_{i-1,\sigma^{\prime }}+(1-\omega_{i,\sigma^{\prime }}^{+}-\omega_{i,\sigma^{\prime }}^{-})\varphi_{i,\sigma^{\prime }}],$$

subject to the boundary conditions *φ*_0,*σ*_=0 and *φ*_*N*,*σ*_=1 for all *σ*. Fixation times can be obtained from a similar relation. The unconditional fixation time *t*_*i*,*σ*_ is the time until absorption (either at *i*=0 or at *i*=*N*) if the population starts in state *i* and if the initial state of the environment is *σ*. Similarly, we can introduce the conditional fixation time for a given initial condition; this is an ensemble average of fixation time for trajectories that result in the fixation of the mutant. A procedure for solving equations (3.1) and its analogue for the conditional and unconditional fixation times in switching environments is described in detail in [21].

### 3.2. Sojourn times

We next introduce an additional tool for the analysis of population dynamics in switching environments, and describe the calculation of mean sojourn times. The concept of sojourn times is well known in the context of birth–death processes with absorbing states [35]; they describe the mean time spent in each state before absorption occurs. More precisely, we write *t*_*i*,*σ*;*j*_ for the mean time the population spends in state *j* before absorption, if it is started in state *i* and if the initial state of the environment is *σ*. We stress that no requirement is made for the environment to be in a specific state when the population ‘sojourns’ in *j*. Additionally, for the purposes of unconditional sojourn times, we do not specify whether the dynamics ends in the state with 0 or *N* mutants. The endpoint is relevant for the so-called conditional sojourn times though; we write $t_{i,\sigma ;j}^{⋆}$ for the mean time the population spends in state *j*, if started at *i* and with initial environmental state *σ*, conditioned on fixation of the mutant. One could measure this object in simulations as follows: run a large number of independent realizations, all started from a population with *i* mutants and *N*−*i* wild-types, and in environment *σ*. Then run each of these samples until the mutant has either gone extinct or reached fixation. Only take into account the runs in which fixation occurs. In this sample of fixated trajectories, measure the average time the population has spent in state *j*.

As part of this work we have developed a method for the calculation of conditional and unconditional sojourn times for general birth–death processes with two species and in switching environments. The calculation is broadly based on backward equation techniques [5,21], and relies on ‘return probabilities’, that is the likelihood that the population returns to state *i* at a later time if it is started there. The key novelty here is that also the dynamics of the environment needs to be accounted for. The calculation is lengthy, and we relegate the full mathematical details to the electronic supplementary material. The unconditional and conditional sojourn times discussed in the next section for specific games and environmental processes have been computed from the electronic supplementary material, equations (S27) and (S34), respectively.

## 4. Dynamics of three-player games

Using this analytical approach to calculate the sojourn times in switching environments (see the electronic supplementary material), we can now examine the specific case of three-player games. We notice interesting behaviour in the three-player games which is not found in the two-player case; a minimum in the conditional fixation times with respect to the switching parameters, for example, was not seen in [21]. There is evidence to suggest that these features apply more generally than just to the three-player cases (see the electronic supplementary material). The sojourn times allow us to investigate where the system spends its time and to understand in more detail the mechanistic origin of the observed behaviour.

### 4.1. Notation

We focus on systems in which the environment can take two different states, *σ*=±1. In [21] results for the case of two-player games were described as a function of the switching probabilities of the environmental state (per time step), *p*^+^=*μ*_+→−_ and *p*^−^=*μ*_−→+_. To better reflect the characteristic properties of the switching process, we introduce *T*=1/*p*^+^+1/*p*^−^ and *δ*^+^=*p*^−^/(*p*^−^+*p*^+^), and use these quantities in place of the parameters *p*^±^. The parameter *T* can be interpreted as the average length of time that it takes for the environment to switch from one state to the other, and then back again. We will refer to *T* as the ‘cycle time’ or ‘switching period’, keeping in mind though that the switching between the environments is stochastic so that there are no strictly periodic cycles. The parameter *δ*^+^ is the average proportion of time spent in the environment *σ*=1. The proportion of time spent in environment *σ*=−1 is given by *δ*^−^=*p*^+^/(*p*^+^+*p*^−^)=1−*δ*^+^. In order for 0≤*p*^±^≤1, we require *T*≥2 and 1/*T*≤*δ*^+^≤1−1/*T*. These conditions are easily understood, keeping in mind the discrete-time nature of the dynamics. The typical cycle must be at least two time steps long, and the minimum proportion of time spent in either environment is one time step, i.e. a fraction 1/*T* of the total (average) cycle.

We are most interested in the dynamics of the system when the internal fixed points are neither very close to one another, nor to the absorbing boundaries. Cases where the fixed points are close to one another or to the boundaries exhibit behaviour similar to games where there are only one or no internal fixed points. Novel behaviour is most clearly observed when the internal fixed points are sufficiently isolated, that is when |*x*_1_−*x*_2_|≫1/*N*, *x*_1_,*x*_2_≫1/*N* and (1−*x*_1_),(1−*x*_2_)≫1/*N*, and so we focus on this regime.

In the following, we will mostly use an example in which the two internal fixed points are located at *x*^⋆^=0.3 and *x*^⋆^=0.7, and with deterministic flow as shown in figure 1. We have tested other cases and have observed similar behaviour. Unless specified otherwise, we always use a population size of *N*=50, and start with one single mutant *i*=1.

### 4.2. Conditional fixation time

**Figure 2.**
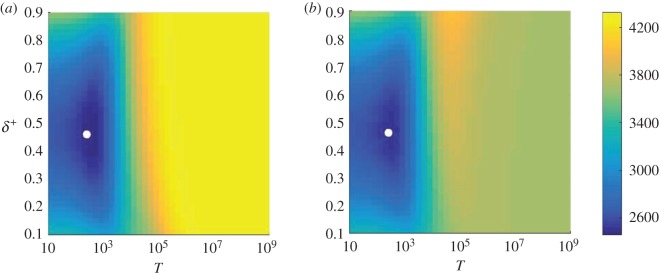
Conditional fixation time as a function of the switching parameters *T* and *δ*^+^. Starting environment is *σ*=1 in (*a*) and *σ*=−1 in (*b*). The fixed points of the system are at *x*^⋆^=0.3 and *x*^⋆^=0.7. There exists a set of switching parameters which minimizes the conditional fixation time. By using sojourn times to reveal where the system spends most of its time, we can understand the origin of this behaviour. Filled white dots in each panel indicate the point at which the conditional fixation time is minimal. These results were obtained using the expressions for the conditional fixation times in [21].

**Figure 3.**
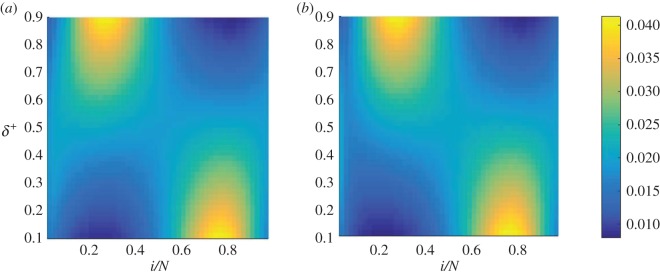
Rescaled conditional sojourn time as a function of the switching parameter *δ*^+^ and the position *x*=*i*/*N*. Specifically, we plot the fraction of time spent in each state, i.e. the sojourn times have been normalized to sum to unity for fixed *δ*^+^. Starting environment *σ*=1 (*a*) and *σ*=−1 (*b*). We have fixed *T*=100. When *δ*^+^∼1 or *δ*^+^∼0, the system tends to loitre around the stable fixed point in the *σ*=1 and *σ*=−1 environments, respectively. These results were calculated using the electronic supplementary material, equation (S34). When *δ*^+^ is close to 0 or 1, the system tends to loitre around the stable fixed point in the predominant environment. When the system spends roughly equal times in either environment, the system is less inclined to loitre around the fixed points, and therefore fixates more quickly.

The conditional fixation time is obtained using the formalism of [21], and is shown as a function of the switching parameters *T* and *δ*^+^ in figure 2. The data in the figure reveal that there is a particular set of switching parameters *T* and *δ*^+^ which minimizes the conditional fixation time, as indicated by filled circles in figure 2. Additional calculations not shown here suggest that this global minimum is no longer present if there are less than two internal fixed points, even in a three-player game. The outcome is effectively very similar to that of a two-player game with one internal fixed point. To investigate the details of the dynamics leading to this effect, it is useful to discuss the conditional sojourn times, to gain an understanding of where the population spends its time on the way to fixation. These are obtained using the theory detailed in the electronic supplementary material and the results are shown in figure 3.

We first focus on the effects of varying the model parameter *δ*^+^ (or conversely *δ*^−^=1−*δ*^+^), which reflect the proportion of time spent in each of the two environmental states. As shown in figure 3, successful trajectories (i.e. those in which the mutant reaches fixation) spend most of their time around the stable fixed point of the ‘dominant’ environment, when either *δ*^+^≫*δ*^−^, or vice versa. For example, when *δ*^+^ is close to one, most time is spent near the stable fixed point *x*^⋆^=0.3 of environment *σ*=1. This illustrates that successful trajectories will loitre around the relevant stable fixed point if one environmental state is significantly more frequent than the other. However, as the parameters *δ*^+^ and *δ*^−^ are moved away from the extremes, the time the system spends in the different states, i=1,…,N=1, is more evenly distributed, and the population has a lower propensity to get trapped near fixed points. At the value of *δ*^+^ which corresponds to the minimum in the conditional fixation time (figure 2), the conditional sojourn times are fairly evenly spread across *i* (figure 3). Trajectories then do not loitre around any of the internal fixed points, and successful runs reach fixation relatively quickly.

**Figure 4.**
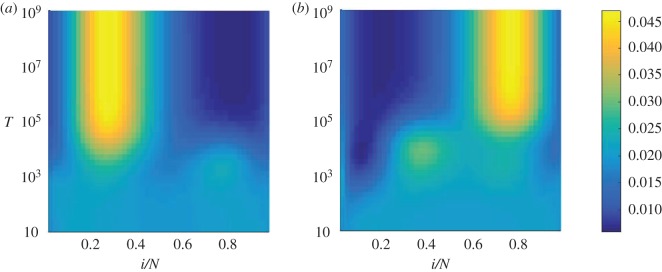
Rescaled conditional sojourn time as a function of the switching parameter *T* and the position *x*=*i*/*N*. Normalization is as in the previous figure. Starting environment *σ*=1 (*a*) and starting environment *σ*=−1 (*b*). We have fixed *δ*^+^=0.5. These results were calculated using the electronic supplementary material, equation (S34). When the environment is switching infrequently, the system has a tendency to loitre around the stable fixed point in the predominant environment. When the switching is frequent, the system seems to spend its time evenly over the range of *x*, but will be less likely to have a direct trajectory towards fixation, since the flow will be changing direction constantly. Balancing these two effects minimizes the conditional fixation time.

We next turn to the effects of varying the timescale of the environmental switching time, i.e. the role of the parameter *T*. When the cycle period *T* is small, the switching process between the environments is fast and so the initial state of the environment at the start of the dynamics does not have any significant effect. This is confirmed in figure 4, where we show the conditional sojourn time as a function of *i*/*N* and *T* for fixed $\delta^{+}=\delta^{-}=\frac{1}{2}$. The two panels in the figure show the data for starts in the two different environments, and the sojourn times are seen to be quantitatively similar across the two panels when *T* is small ($T≲10^{3}$ in our example). The sojourn time is then relatively constant across different states *i* of the population, indicating that time is spent fairly evenly; we note again that the data in the figure are for *δ*^+^=0.5. This indicates that the population does not have sufficient time to settle near either of the fixed points, owing to the fast switching dynamics. While each fixed point is attractive in one environment and a repeller in the other, these effects ‘average’ out under fast environmental switching.

For longer duration of the switching cycle *T*, however, the system begins to spend longer stretches of time in either environment, and so is able to spend more time at either fixed point. The conditional sojourn times begin to peak around the locations of the fixed points for $T≳10^{3}$ (figure 4). If the cycle is longer still ($T≳10^{4}$), most of the dynamics occurs in the starting environment, and hence the population will spend most of its time around the stable fixed point in that environment. This is where we begin to see a marked difference between the two panels in figure 4. For timescales above *T*≈10^5^, the environment effectively never switches state before fixation is reached; this corresponds to the regime in figure 2 in which the conditional fixation time reaches a value which is characteristic of the starting environment and independent of *δ*^+^.

The value of the cycle period *T*∼10^3^ which minimizes the conditional fixation time in figure 2 corresponds roughly to the point at which the environment is neither switching so often that the trajectories are constantly changing direction, nor switching so little that the system loitres around a fixed point. This can be seen in figure 4 as the value of *T* where the sojourn times begin to stop being spread equally across all values of *i*/*N*, and start to peak around both fixed points. This can be likened to a resonance effect noting that the fixation times are also of order 10^3^ (e.g. figure 2).

We note that a similar minimum in the conditional fixation times can be observed in five-player games, but not in four-player games. We discuss this further in the electronic supplementary material.

### 4.3. Probability for a single mutant to reach fixation

**Figure 5.**
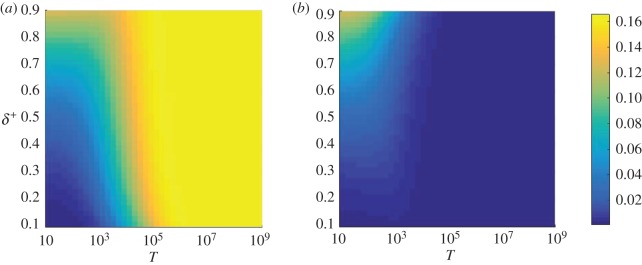
Fixation probability as a function of the switching parameters *T* and *δ*^+^. Starting environment *σ*=1 (*a*) and *σ*=−1 (*b*). Broadly speaking, the greater the amount of time the system spent in environment *σ*=1, the greater is the fixation probability. These results were obtained using the formulae for the conditional fixation times provided in [21]. As *T* is increased past the typical fixation time, the system will spend most of its time in the starting environment. We note that fixation probability increases with *T* in the *σ*=1 starting environment and decreases with *T* in the *σ*=−1 starting environment. We also note that the fixation probability increases with *δ*^+^ in both starting environments. Therefore, the likelihood of fixation appears to be only related to how much time the system spends in the *σ*=1 environment. We discuss why this might be using the sojourn times (see main text).

The probability that a single mutant reaches fixation is shown in figure 5 as a function of the switching period, *T*, and the fraction of time spent in environment *σ*=1, *δ*^+^. The two panels show data for starts in either of the environmental states. The figure reveals that there is no combination of *T* and *δ*^+^, which would extremize the fixation probability in the same way as the conditional fixation times. The main conclusion we draw from the figure is, quite simply, that the likelihood that the mutant fixates is greater the higher the proportion of time that the system spends in environment *σ*=+1, corresponding to the flow depicted in figure 1*a*. This is the case for starts in either of the two environmental states. We also note that the fixation probability increases with the cycle period *T* if the start occurs in environment *σ*=1, but that it decreases with *T* for starts in *σ*=−1.

**Figure 6.**
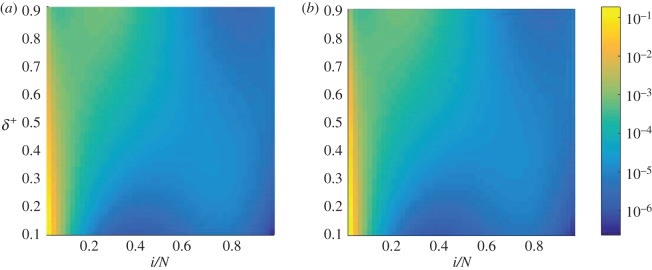
Unconditional sojourn time as a function of the switching parameter *δ*^+^ and the position *x*=*i*/*N*. Starting environment *σ*=1 (*a*) and *σ*=−1 (*b*). Data are for fixed *T*=100. The system can be seen to spend less time around the initial position at *i*=1 as *δ*^+^ is increased, indicating that the system leaves the starting position more easily when *δ*^+^ is higher, as one might expect. This correlates with the increase in fixation probability with *δ*^+^ in figure 5. The qualitative behaviour is roughly the same in either environment. This suggests that the fixation probability is mostly governed by how likely the mutants are to perish at the beginning of the process. These results were calculated using the electronic supplementary material, equation (S27).

**Figure 7.**
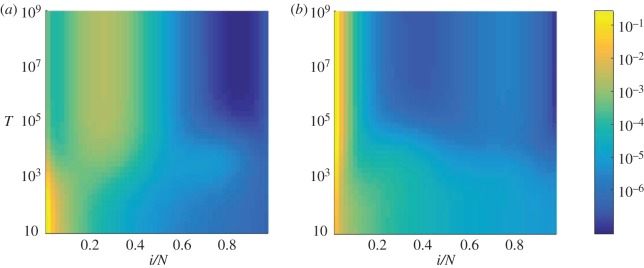
Unconditional sojourn times as a function of the switching parameter *T* and the position *x*=*i*/*N*. Starting environment *σ*=1 (*a*) and *σ*=−1 (*b*), for fixed *δ*^+^=0.5. As *T* is increased, the system spends more time in the starting environment. In (*a*), the system moves away from the starting position more easily with increasing *T*, which corresponds to the increase with *T* of the fixation probability in figure 5*a*. The opposite is true in (*b*). The fixation probability can be seen to decrease with *T* in figure 5*b*. This again suggests that the fixation probability is mostly governed by how likely the mutants are to perish at the beginning of the process. These results were calculated using the electronic supplementary material, equation (S27).

We now comment on the mechanisms generating these effects. The system always begins with one mutant, very close to the absorbing boundary at *i*=0. One might expect, therefore, that the propensity of the system to move away from the initial position, and from extinction, would have a great impact on the overall fixation probability. The unconditional sojourn times shown in figure 6 confirm that the system spends less of its trajectory near the starting position as *δ*^+^ is increased. This correlates with the increase in fixation probability with *δ*^+^ in figure 5. In figure 7, we show the unconditional sojourn time as a function of the cycle period *T*. As this timescale *T* is increased, the initial state of the environment becomes more relevant, as the first switch occurs later on average. This reduces the probability of immediate extinction for starts in the environment *σ*=1 for which the flow is away from the *i*=0 boundary. This correlates with the increase in fixation probability with *T* in figure 5*a*. If the initial state of the environment is *σ*=−1, the argument is reversed. In this environment the flow is towards *i*=0 for small mutant numbers; a prolonged initial time spent in this environment hence reduces the likelihood that the mutant is successful, as indicated by the decrease of fixation probability in figure 5*b*.

We have also examined games with three or four internal fixed points (that is, four- or five-player games), and we find very similar qualitative behaviour in the fixation probabilities. These results are provided in the electronic supplementary material.

### 4.4. Fixation in finite time

**Figure 8.**
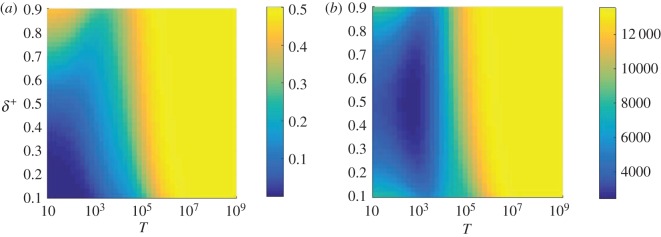
Fixation probability (*a*) and conditional fixation time (*b*) as a function of the switching parameters *δ*^+^ and *T* for the case *β*=1. In both cases, the starting environment is *σ*=1 and the fixed points are located at *x*^⋆^=0.3 and *x*^⋆^=0.7. The behaviour is qualitatively the same as in figures 2 and 5.

The combination of figures 2 and 5 reveals an intriguing observation. The fixation time, conditioned on fixation of the mutant, is minimal for a certain combination of *T* and *δ*^+^, as indicated in figure 2. On the other hand, no such extremum is found for the fixation probability in figure 5. The fixation probability measures the likelihood for the mutant to be successful eventually—including at long times. This indicates an interesting balance of two effects: if evolution is allowed to run indefinitely the mutant has the highest chance of success when *T* and *δ*^+^ are large, and the starting environment is *σ*=1. If however, we are interested in fixation on moderate timescales, the mutant does better near the point of minimal conditional fixation time in figure 2. To investigate this further, we focus on a case with relatively strong selection, *β*=1. As is exemplified in figure 8, varying *β* does not appear to have a very profound effect on the qualitative behaviour of the system, but in this case it does accentuate the features at which we are looking. The corresponding conditional fixation times and fixation probabilities are shown as functions of *δ*^+^ and *T* in figure 8. Again, a minimum in fixation time is found, but no extremum of the fixation probability.

We introduce *Q*_*i*,*σ*_(*t*) as the probability that the mutant has reached fixation *t* time steps (or sooner) after the system is started with *i* mutants and in environmental state *σ*. We then have the discrete-time backward master equation (see e.g. [5,6,35] for general references)

4.1 $$Q_{i,\sigma }(t+1)=\sum_{\sigma^{\prime }}\mu_{\sigma \to \sigma^{\prime }}[\omega_{i,\sigma }^{+}Q_{i+1,\sigma^{\prime }}(t)+\omega_{i,\sigma }^{-}Q_{i-1,\sigma^{\prime }}(t)+(1-\omega_{i,\sigma }^{+}-\omega_{i,\sigma }^{-})Q_{i,\sigma^{\prime }}(t)].$$

The initial conditions are *Q*_*i*,*σ*_(*t*=0)=*δ*_*i*,*N*_ for all *σ*, and allow us to numerically obtain the *Q*_*i*,*σ*_(*t*) by iterating equation (4.1) forwards.

**Figure 9.**
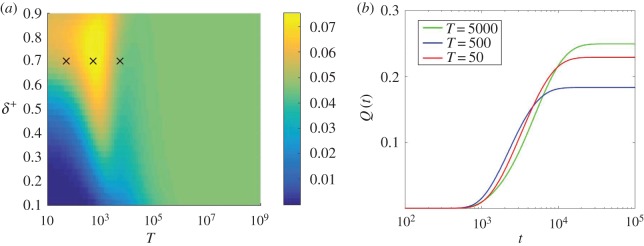
(*a*) Proportion of trajectories which have reached state *i*=*N* by time *t*=2000. (*b*) Fraction of trajectories which have reached *i*=*N* by time *t*. We fix *δ*^+^=0.7 and show three different choices of *T* in (*b*); these are indicated by the crosses in (*a*). This demonstrates the balance between high fixation probability and low conditional fixation time. At *t*=2000, the set of switching parameters which gives the highest amount of fixated trajectories is neither the set with the lowest associated fixation probability nor the set with the lowest associated conditional fixation time. All data are for *β*=1, starting at *i*=1 and in environment *σ*=1.

Results are summarized in figure 9. In figure 9*a*, we show the fraction of samples, started with one single mutant, in which the mutant has reached fixation by time *t*=2000 and a maximum is discernible near *δ*^+^=0.7 and just below *T*≈10^3^. This demonstrates that the total fraction of samples that reach fixation by a finite time *t* can have a local maximum at a location in the *δ*^+^−*T* plane, even when the eventual fixation probability does not have such a maximum (cf. figure 8*a*). This is clarified further in figure 9*b*, where we show *Q*_1,*σ*=1_(*t*) as a function of time, *t*, for three different choices of the cycle period (and at a fixed value of *δ*^+^=0.7). These correspond to the points marked in figure 9*a*. While more trajectories reach fixation eventually for *T*=5000, the proportion that have reached fixation at times *t*≈1000−5000 is higher for the other two choices of *T* shown in the figure (*T*=50 and *T*=500).

## 5. Conclusion and outlook

In summary, we have analysed the dynamics of fluctuating multiplayer games in finite populations. Fluctuations of the pay-off matrix are taken to originate from changes of an environmental state affecting the relative success of mutants and resident wild-types. These environmental fluctuations could, for example, represent availability or the absence of nutrients, the application of treatment in the context of bacterial populations or variations in other external conditions. Our analysis focuses on a stylized model with interaction between multiple individuals—modelled as a multiplayer game—but representing general nonlinear pay-off structures. This adds complexity relative to the two-player case studied in [21]. In this earlier work, fitness differences between mutants and wild-types are linear in their relative frequencies, and hence at most one internal equilibrium point is permitted by the resulting replicator equations. In this work, we address higher-order interaction leading to more complex frequency-dependent fitness landscapes, with multiple non-trivial selection balance points. Specifically, we focus on games which allow two internal fixed points and on the regime in which these are well separated from each other and from the states at which mutants have reached fixation or gone extinct. We have then analysed in detail the likelihood for an invading mutant to reach fixation in the resident population. We have also studied the dynamics leading to fixation, in particular conditional fixation times, and the time spent in each state of the population on the path to fixation, the so-called sojourn times [35]. We have presented a comprehensive theory to calculate these analytically for multiplayer games in finite populations subject to fluctuating environments with discrete states. A way of perhaps simplifying the fairly obtuse theoretical expressions for the sojourn times may be to use an asymptotic expansion in terms of the system size, as mentioned in [36,37].

We have only discussed the case of two environments in this paper, but the theory in the electronic supplementary material does apply to an arbitrary number of environmental states. An interesting extension to this work could be in relating the limit of infinite discrete environmental states to continuous environmental noise and the results of models such as those used in [19,20]. In their analysis of a Prisoners’ Dilemma game with continuously fluctuating selection strength, the authors of [19], for example, find non-monotone behaviour of the mean fixation time and of the fixation probability as a function of the correlation time of the environmental noise. It would be interesting to investigate in more detail whether the unbounded range of the extrinsic noise used in [19] is a key factor for this behaviour.

Our analysis indicates that the timescales and detailed dynamics of the environmental switching process can affect the success of the invading mutant in several ways. For example, we find that the conditional fixation time can have a local minimum in the space of all (Markovian) switching processes, indicating that the mutant succeeds quickly under those circumstances, if it reaches fixation. At the same time the fixation probability appears to exhibit monotonous behaviour in the timescale of the switching process and fraction of time spent in either of two environments. This indicates an interesting balance of two effects: a propensity to reach fixation quickly and the overall fixation probability at long times. A more detailed analysis of the dynamics, based on a backward-master equation approach, reveals that these effects may be in competition with each other. There are switching dynamics for which there is a pronounced tendency to reach fixation in the early stages of the dynamics, but other parameters of the environmental dynamics may lead to a higher chance of fixation eventually.

In this paper, we have largely focused on the effect that a fluctuating external environment has on the success of mutants, where we had a fixed pay-off matrix in either environment. We might also have set ourselves the task of finding the optimal pay-off matrices and/or arrangement of fixed points for the success of the mutant. That is, we could have inquired as to the optimal nature of the mutant in a game with given switching dynamics, and compared this to the non-switching case. Obviously, there is always a trivial answer to this question (a mutant which is dominant in all states of the environment), and hence to make the question have meaning, certain constraints on the abilities of the mutant would have to be imposed. What these constraints should be would very much depend on the specific biological context. We believe this discussion is beyond the remit of this paper but could be an interesting avenue of further research. Other extensions might include scenarios in which the environmental dynamics is coupled to the state of the population, that is the environmental switching rates depend on the composition of the population.

In broader terms, our findings contribute to constructing a more general theory of population dynamics with selection, random genetic drift and environmental fluctuations. While we have focused on a selected set of three-player games, the calculation of sojourn times in switching environments is applicable to general birth–death processes with fluctuating discrete environmental states. Examples of types of biological systems where our work may be applicable have been examined in [38,39]. Multiplayer games include in particular biological auctions or markets, and situations in which there is competition for a public good. An overview of applications of multiplayer games in biology and the social sciences can be found in [33,40]. Hamilton has characterized the complexity of multiplayer games as ‘sea-sickness’ [22]. We believe our work is a contribution to reducing this discomfort in dealing with multiplayer dynamics and towards an understanding of the outcomes of evolutionary processes with fluctuating non-linear interaction.

## Supplementary Material

Supplementary material

## Data accessibility

The computer codes used to produce the data in the figures are available at http://dx.doi.org/10.17632/j4rzkgvdjb.1.
