# Supplementary material for: How successful are mutants in multiplayer games with fluctuating environments? Sojourn times, fixation and optimal switching
Source: R Soc Open Sci. 2018 Mar 21;5(3):172176. doi: 10.1098/rsos.172176 (PMC5882734; doi:10.1098/rsos.172176)
Supplement: Supplementary material [file rsos172176supp1.pdf]

How successful are mutants in multi-player games with fluctuating environments? Sojourn times, fixation and optimal switching

Supplementary Material

Submitted to Royal Society Open Science

Joseph W. Baron* and Tobias Galla †

Theoretical Physics, School of Physics and Astronomy,
The University of Manchester, Manchester M13 9PL, United Kingdom

January 28, 2018

1 Construction of multi-player games

We briefly describe the construction of multi-player games with specified locations of internal fixed points in the deterministic limit. In this limit the dynamics are given by

$$\dot{x} = \omega_\sigma^+(x) - \omega_\sigma^-(x). \tag{S1}$$

The subscript σ is not relevant for the argument that follows, so we omit it for the remainder of this section of the Supplement. Given that each of the rates contains a factor $x(1-x)$ there are always two trivial fixed points $x^\star = 0$ and $x^\star = 1$. The remaining fixed points of Eq. (S1) are the – non-trivial – roots of $\omega^+(x) - \omega^-(x) = 0$. This is equivalent to

$$\pi_A(x) = \pi_B(x), \tag{S2}$$

where the latter payoffs are given by

$$\begin{aligned} \pi_A(x) &= \sum_{j=0}^{n-1} a_j \binom{n-1}{j} x^j (1-x)^{n-j-1} \\ \pi_B(x) &= \sum_{j=0}^{n-1} b_j \binom{n-1}{j} x^j (1-x)^{n-j-1} \end{aligned} \tag{S3}$$

*joseph.baron@student.manchester.ac.uk
†tobias.galla@manchester.ac.uk

in an n-player game. We note that $\left(\begin{array}{c} n-1 \\ j \end{array}\right) x^j(1-x)^{n-j-1}$ is the probability for a given player to face j opponents of type A and $n-1-j$ opponents of type B in an n-player game, if the fraction of type A-players in the population is x.

1.1 General construction

The construction of the game with specified non-trivial fixed points $x_1^\star, \ldots, x_{n-1}^\star$ is equivalent to finding coefficients $c_j \equiv a_j - b_j$, $j = 0, 1, \ldots, n-1$, such that

$$\sum_{j=0}^{n-1} c_j \left(\begin{array}{c} n-1 \\ j \end{array}\right) x^j(1-x)^{n-j-1} = 0 \tag{S4}$$

for $x \in \{x_1, \ldots, x_{n-1}\}$.
We construct a solution by induction. The problem is straightforward for $n = 2$, in which case it reduces to finding c_0 and c_1 such that $c_0(1 - x_1^\star) + c_1 x_1^\star = 0$. One finds

$$\frac{c_1}{c_0} = 1 - \frac{1}{x_1^\star}. \tag{S5}$$

Suppose now that we have constructed $c_0, \ldots, c_{n-1}$ such that $x_1^\star, \ldots, x_{n-1}^\star$ are the roots of

$$\sum_{j=0}^{n-1} c_j \left(\begin{array}{c} n-1 \\ j \end{array}\right) x^j(1-x)^{n-j-1} = 0. \tag{S6}$$

We can introduce a new root at $x_n^\star$ by multiplying by $1 - x + \left(1 - \frac{1}{x_n^\star}\right) x$. One then obtains

$$\begin{aligned} &\sum_{j=0}^{n-1} \left[c_j \left(\begin{array}{c} n-1 \\ j \end{array}\right) + \left(1 - \frac{1}{x_n^\star}\right) c_{j-1} \left(\begin{array}{c} n-1 \\ j-1 \end{array}\right)\right] x^j \left(1-x\right)^{n-j} \\ &+ c_0 \left(1-x\right)^n + c_{n-1} \left(1 - \frac{1}{x_n^\star}\right) x^n = 0. \end{aligned} \tag{S7}$$

This can be written in the form

$$\sum_{j=0}^{n} c'_j \left(\begin{array}{c} n \\ j \end{array}\right) x^j(1-x)^{n-j} = 0. \tag{S8}$$

with

$$\begin{aligned} c'_0 &= c_0, \\ c'_n &= c_{n-1} \left(1 - \frac{1}{x_n^\star}\right), \\ c'_j &= \frac{1}{\left(\begin{array}{c} n \\ j \end{array}\right)} \left[c_j \left(\begin{array}{c} n-1 \\ j \end{array}\right) + c_{j-1} \left(1 - \frac{1}{x_n^*}\right) \left(\begin{array}{c} n-1 \\ j-1 \end{array}\right)\right]. \end{aligned} \tag{S9}$$

This completes the inductive construction.

1.2 Three-player games

The resulting relations are relatively compact for three-player games. We will have internal fixed points at $x_1^\star$ and $x_2^\star$ if we choose

$$\frac{c_{1,\sigma}}{c_{0,\sigma}} = \frac{1}{2}\left[\left(1-\frac{1}{x_1^\star}\right)+\left(1-\frac{1}{x_2^\star}\right)\right], \tag{S10}$$

and

$$\frac{c_{2,\sigma}}{c_{0,\sigma}} = \left(1-\frac{1}{x_1^\star}\right)\left(1-\frac{1}{x_2^\star}\right). \tag{S11}$$

We have here included the subscript σ to indicate the dependence of the game on the environmental state. The coefficient $c_{0,\sigma}$ can be chosen arbitrarily, its sign determines the direction of the flow at $x=0$, and hence the stability of $x_1^\star$ and $x_2^\star$, respectively. In the main paper we use $c_{0,\sigma=1}=1$ and $c_{0,\sigma=-1}=-1$.

2 Analytical calculation of sojourn times

The sojourn time is the mean amount of time that the system spends in any one position i, given the starting position and environment. Both conditional (only trajectories which result in fixation contribute to the average) and unconditional sojourn times can be calculated. In the following subsections, we go about deriving expressions for the sojourn times in fluctuating environments. We note that the following discussion is only directly applicable to well-mixed populations with two species (strategies), but it would also be possible, in theory, to formulate the problem more generally. Obtaining closed-form solutions to this more general problem would be distinctly more complicated as the solution in the 2-species case relies on the use of telescopic sums. Another simplification we have made is the assumption that the environmental switching probability is independent of the state i of the population. The mathematical procedure is still possible without making such an assumption but things do simplify in our case.

2.1 Initial steps of the calculation

In order to compute sojourn times we introduce the quantity

$$\varphi_{i,\sigma;j,\sigma'} = \text{Prob}\left(\begin{array}{l|l} \text{There exists a time } t\geq t_0 \text{ at which the system} & \text{The population is started in state} \\ \text{reaches state } j\text{, and when it does so for the} & i \text{ and the environent in } \sigma \text{ at ini-} \\ \text{first time the environment is in state } \sigma'. & \text{tial time } t_0. \end{array}\right). \tag{S12}$$

We note that the initial time t_0 is obviously immaterial, as the dynamics is Markovian. One then has

$$\varphi_{i,\sigma;j,\sigma'} = \sum_{\sigma''}\mu_{\sigma\to\sigma''}\left[\omega^+_{i,\sigma}\varphi_{i+1,\sigma'';j,\sigma'}+\omega^-_{i,\sigma}\varphi_{i-1,\sigma'';j,\sigma'}+(1-\omega^+_{i,\sigma}-\omega^-_{i,\sigma})\varphi_{i,\sigma'';j,\sigma'}\right], \tag{S13}$$

with the following boundary conditions

$$\varphi_{0,\sigma;j,\sigma'}=\delta_{0,j}\delta_{\sigma,\sigma'},\ \varphi_{N,\sigma;j,\sigma'}=\delta_{N,j}\delta_{\sigma,\sigma'},\ \varphi_{j,\sigma;j,\sigma'}=\delta_{\sigma,\sigma'}. \tag{S14}$$

The first and second of these reflect the fact that the states $i=0$ and $i=N$ are absorbing, so once the population is in state $i=0$ or $i=N$ it remains there at all future times. In the third relation in Eq. (S14) we have $i=j$ so that trajectories, started at (i,σ), will reach state j immediately at $t=t_0$; they then only contribute to the above probability if $\sigma'=\sigma$.

For a fixed j Eqs. (S14) impose constraints on $\varphi_{i,\sigma;j,\sigma'}$ at $i=0$, $i=N$ and at $i=j$. Focusing on a given value of j it is hence convenient to treat the cases $i<j$ and $i>j$ separately. To proceed we introduce the following quantities,

$$\psi_{i,\sigma;j,\sigma'} = \sum_{\sigma''} \mu_{\sigma\to\sigma''}\varphi_{i,\sigma'';j,\sigma'}. \tag{S15}$$

Focusing first on $j>i$, we define

$$\nu_{i,\sigma';j,\sigma'} = \psi_{i,\sigma;j,\sigma'} - \psi_{i-1,\sigma;j,\sigma'}, \tag{S16}$$

and we then arrive at the following after substitution in Eq. (S13),

$$\nu_{i+1,\sigma;j,\sigma'} = \gamma_{i,\sigma}\nu_{i,\sigma;j,\sigma'} + \frac{1}{\omega^+_{i,\sigma}}\left[\left(\underline{\underline{\mu}}^{-1} - \mathbb{1}\right)\sum_{k=1}^{j}\underline{\nu}_{k,\sigma;j}\right]_{\sigma'}. \tag{S17}$$

We have used vector and matrix notation for convenience. Indices run over the states of the environment; for example the components of $\underline{\nu}_{k,\sigma;j}$ correspond to the index σ', and $\underline{\underline{\mu}}$ has entries $\mu_{\sigma\to\sigma'}$.
We then use the condition

$$\sum_{i=1}^{j}\nu_{i,\sigma;j,\sigma'} = \mu_{\sigma\to\sigma'} \tag{S18}$$

to determine $\nu_{i,\sigma;j,\sigma'}$. The probabilities $\varphi_{i,\sigma;j,\sigma'}$ can then be found using

$$\underline{\varphi}_{i,\sigma;j} = \underline{\underline{\mu}}^{-1}\sum_{k=1}^{i}\underline{\nu}_{k,\sigma;j}. \tag{S19}$$

Similarly, for $j<i$ we write $\lambda_{i,\sigma;j,\sigma} = \psi_{i,\sigma;j,\sigma} - \psi_{i+1,\sigma;j,\sigma}$, and find

$$\lambda_{i-1,\sigma;j,\sigma'} = \frac{1}{\gamma_{i,\sigma}}\lambda_{i,\sigma;j,\sigma'} + \frac{1}{\omega^-_{i,\sigma}}\left[\left(\underline{\underline{\mu}}^{-1} - \mathbb{1}\right)\sum_{k=i}^{N-1}\underline{\lambda}_{k,\sigma;j}\right]_{\sigma'}, \tag{S20}$$

One then proceeds using $\sum_{i=j}^{N-1}\lambda_{i,\sigma;j,\sigma'} = \mu_{\sigma\to\sigma'}$, and $\underline{\varphi}_{i,\sigma;j} = \underline{\underline{\mu}}^{-1}\sum_{k=i}^{N-1}\underline{\lambda}_{k,\sigma;j}$.
Now we have at our disposal a means by which to calculate the complete set of probabilities $\varphi_{i,\sigma;j,\sigma'}$. Using these probabilities, one can then compute the unconditional and conditional sojourn times.

2.2 Calculation of unconditional sojourn times

As the next step we compute

$$r_{i,\sigma;\sigma'} = \text{Prob}\left(\begin{array}{l|l}\text{There exists a time } t>t_0 \text{ at which the} & \text{The population is started in state} \\ \text{population returns to state } i\text{, and when it} & i \text{ and the environent in } \sigma \text{ at ini-} \\ \text{does so for the first time the environment} & \text{tial time } t_0. \\ \text{is in state } \sigma'. & \end{array}\right). \tag{S21}$$

We stress the requirement that t be strictly greater than t_0, marking a difference compare to the above definition of $\varphi_{i,\sigma;j,\sigma'}$, where we only require $t\geq t_0$. Hence, $r_{i,\sigma;\sigma'}$ is in general distinct from $\varphi_{i,\sigma;i,\sigma'} = \delta_{\sigma,\sigma'}$. We have

$$r_{i,\sigma;\sigma'} = \mu_{\sigma\to\sigma'}\left(1-\omega^+_{i,\sigma}-\omega^-_{i,\sigma}\right) + \sum_{\sigma''}\mu_{\sigma\to\sigma''}\left(\omega^+_{i,\sigma}\varphi_{i+1,\sigma'';i,\sigma'} + \omega^-_{i,\sigma}\varphi_{i-1,\sigma'';i,\sigma'}\right). \tag{S22}$$

We can now turn to sojourn times. Consider a trajectory which begins in state (i, σ). The probability of spending a total of t time steps in a particular state j, irrespective of the state the environment is in at that time, is then given by

$$q_t(j|i,\sigma) = \sum_{\sigma_1\ldots\sigma_{t-1}} \varphi_{i,\sigma;j,\sigma_1} r_{j,\sigma_1;\sigma_2} r_{j,\sigma_2;\sigma_3} \ldots r_{j,\sigma_{t-2};\sigma_{t-1}} \left(1 - \sum_{\sigma_t} r_{j,\sigma_{t-1};\sigma_t}\right). \tag{S23}$$

The trajectory first has to reach state j, as indicated by $\varphi_{i,\sigma;j,\sigma_1}$, it then has to 'return' t times [in the sense of Eq. (S22)], indicated by the factors $r_{j,\sigma_1;\sigma_2} r_{j,\sigma_2;\sigma_3} \ldots r_{j,\sigma_{t-2};\sigma_{t-1}}$, and it must then not return to j again, see the factor $1 - \sum_{\sigma_t} r_{j,\sigma_{t-1};\sigma_t}$. This can be written in a more compact matrix notation

$$\underline{q_t}(j|i) = \underline{\underline{\varphi_{ij}}} \left(\underline{\underline{r_j}}\right)^{t-1} \underline{x_j}, \tag{S24}$$

where

$$\left(\underline{x_j}\right)_\sigma = 1 - \sum_{\sigma'} r_{j,\sigma;\sigma'}. \tag{S25}$$

The unconditional sojourn time is then the first moment of the distribution over t defined by $q_t(j|i,\sigma)$,

$$t_{i,\sigma;j} = \sum_{t=1}^{\infty} t q_t\left(j|i\sigma\right) = \left(\underline{\underline{\varphi_{ij}}} \left[\sum_{t=1}^{\infty} t \left(\underline{\underline{r_j}}\right)^{t-1}\right] \underline{x_j}\right)_\sigma. \tag{S26}$$

Letting $\underline{\underline{\mathbb{1}}} - \underline{\underline{r_j}} = \underline{\underline{S_j}}$, one can evaluate the series to find

$$t_{i,\sigma;j} = \left(\underline{\underline{\varphi_{ij}}} \left(\underline{\underline{S_j^{-1}}}\right)^2 \underline{x_j}\right)_\sigma. \tag{S27}$$

Therefore, one can calculate the unconditional Sojourn times once the probabilities $\underline{\underline{\varphi_{ij}}}$ have been obtained as described above.

2.3 Conditional sojourn times

The conditional Sojourn times can be calculated in a similar way. We introduce the following shorthand

$$q_t^*\left(j|i,\sigma\right) = \text{Prob}\left(\begin{array}{l}\text{The population spends exactly } t \\ \text{steps at } j \text{ before absorption.}\end{array}\middle| \begin{array}{l}\text{The starting point is } (i,\sigma)\text{, and} \\ \text{the mutant reaches fixation.}\end{array}\right). \tag{S28}$$

Using Bayes' theorem we have

$$q_t^*\left(j|i,\sigma\right) = \frac{\text{Prob}\left(\text{spends } t \text{ steps at } j \text{ and reaches fixation} \mid \text{starts at } (i,\sigma)\right)}{\text{Prob}\left(\text{reaches fixation} \mid \text{starts at } (i,\sigma)\right)}. \tag{S29}$$

We also note that

$$\text{Prob}\left(\text{reaches fixation} \mid \text{starts at } (i,\sigma)\right) = \varphi_{i,\sigma} = \sum_{\sigma'} \varphi_{i,\sigma;N,\sigma'}. \tag{S30}$$

Hence, similar to the unconditional case,

$$q_t^*\left(j|i,\sigma\right) \sum_{\sigma'} \varphi_{i,\sigma;N,\sigma'} = \sum_{\sigma_1\ldots\sigma_{t-1}} \varphi_{i,\sigma;j,\sigma_1} r_{j,\sigma_1;\sigma_2} r_{j,\sigma_2;\sigma_3} \ldots r_{j,\sigma_{t-2};\sigma_{t-1}} \left(\underline{y_j}\right)_{\sigma_{t-1}}, \tag{S31}$$

where

$$\left(\underline{y_j}\right)_\sigma = \omega^+_{j,\sigma} \sum_{\sigma_t} \mu_{\sigma\to\sigma_t} \left(1 - \sum_{\sigma'} \varphi_{j+1,\sigma_t;j,\sigma'}\right). \tag{S32}$$

Using more compact notation we have

$$q^*_t\left(j|i,\sigma\right) = \frac{\left(\underline{\underline{\varphi_{ij}}}\left(\underline{\underline{r_j}}\right)^{t-1}\underline{y_j},\right)}{\sum_{\sigma'} \varphi_{i,\sigma;N,\sigma'}}, \tag{S33}$$

Using a procedure similar to the unconditional case, we obtain

$$t^*_{i,\sigma;j} = \sum_{t=1}^{\infty} t q^*_t\left(j|i,\sigma\right) = \frac{\left(\underline{\underline{\varphi_{ij}}}\left(\underline{\underline{S_j^{-1}}}\right)^2 \underline{y_j}\right)_\sigma}{\sum_{\sigma'} \varphi_{i,\sigma;N,\sigma'}}. \tag{S34}$$

These conditional Sojourn times can also be calculated, given the complete set of probabilities $\varphi_{i,\sigma;j,\sigma'}$.

3 n-Player games ($n > 3$)

We have discussed in the main part of the paper the case of 3-player games, which have 2 internal fixed points, and we have contrasted this with the case of 2-player games, which have 1 internal fixed point. One might well wonder about the observed phenomena in games with more than 3-players. In Figs. S2 and S3 we show the fixation probabilities and conditional fixation times for 4- and 5-player games respectively. We also include the corresponding plots for 2-player games in Fig. S1 for comparison. The qualitative behaviour of the fixation probabilities is the same in the 2-, 3-, 4- and 5-player cases.

The conditional fixation times for the 2- and 4-player cases are very similar to each other, as are those for the 3- and 5-player games, suggesting that it is the pattern of stable and unstable fixed points that primarily determines the behaviour of the conditional fixation times. So, one only observes the minimum in the conditional fixation times when stability of the fixed points at the absorbing boundaries is opposite.

In the cases where the absorbing fixed points are of the same type (both stable, or both unstable), the environment where conditional fixation occurs most slowly is the one where the mutants are also most likely to succeed, $\sigma = 1$. So, spending lots of time in the $\sigma = 1$ environment, where the absorbing boundary at $x = 1$ is repulsive, is bound to make the conditional fixation time larger. Whereas, if the system spends more time in the environment where the boundary at $x = 1$ is stable, only the trajectories which are direct will tend to achieve fixation. So, the $\sigma = -1$ environment is more conducive to a low conditional fixation time when the absorbing boundaries are of opposite type.

In the cases where the absorbing boundaries are of different types, the $\sigma = 1$ environment is both the environment where the boundary at $x = 1$ is attractive and is also the environment where the mutants are most likely to be successful. The mutants will be repulsed by the fixed point at $x = 1$ in the $\sigma = -1$ environment, but they will also be less likely to fixate and thereby contribute to the conditional fixation time. For this reason, neither environment has the lower conditional fixation time outright. Switching between the two environments allows a balance to be struck between these two effects, hence we observe the minimum in the conditional fixation times.

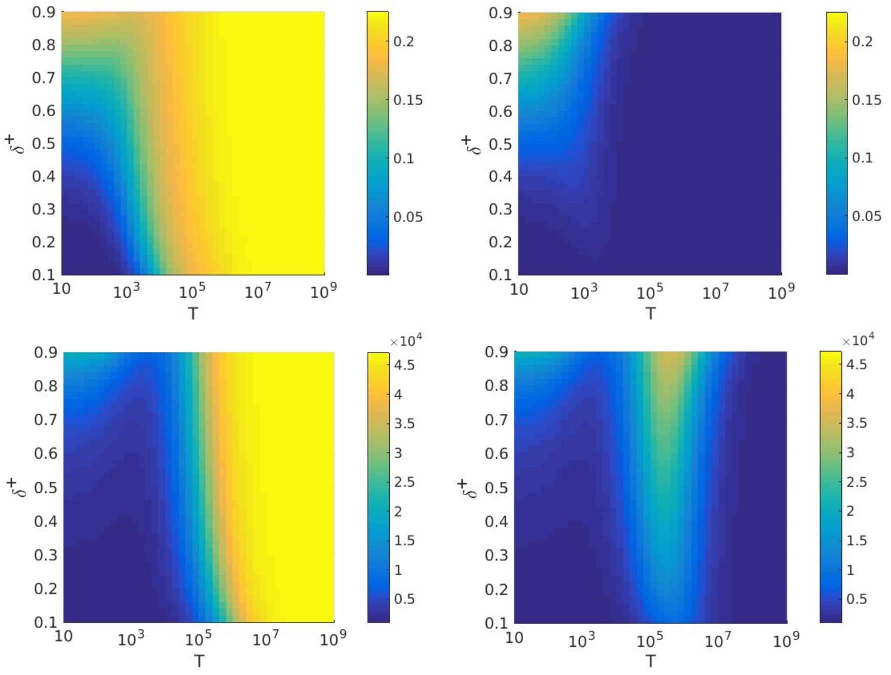

Figure S1. Fixation probabilities (top two panels) and conditional fixation times (bottom two panels) for 2-player games (1 internal fixed point). The staring environment is $\sigma = 1$ for the panels on the left and $\sigma = -1$ for the panels on the right. The fixed point is located at $x = 0.5$. The behaviour of the fixation probabilities is very similar for all the n-player games tested. The behaviour of the conditional fixation times turns out to be very similar to the 4-player case but not to the 3- or 5- player cases.

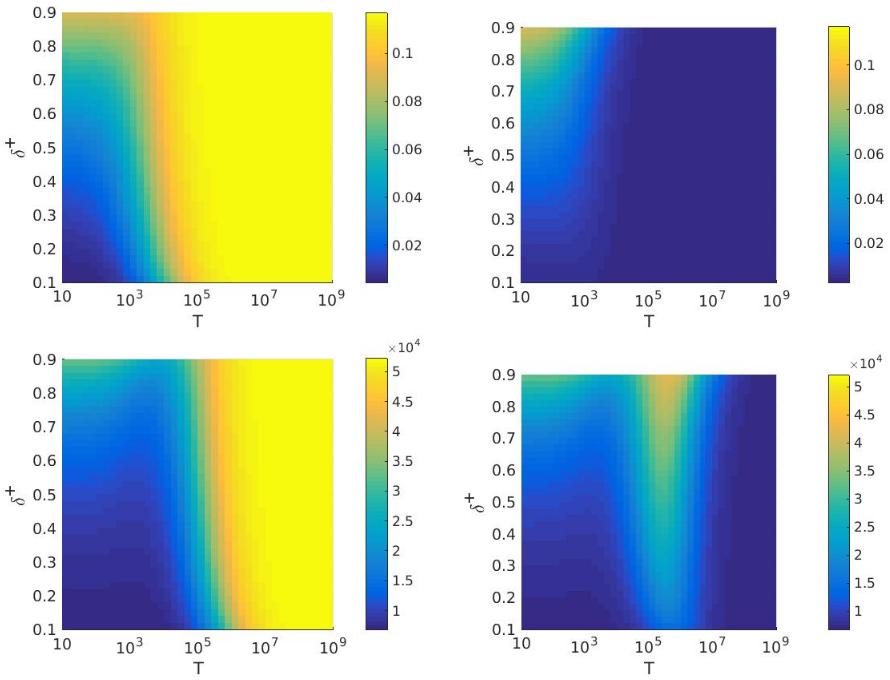

Figure S2. Fixation probabilities (top two panels) and conditional fixation times (bottom two panels) for 4-player games (3 internal fixed points). The staring environment is $\sigma = 1$ for the panels on the left and $\sigma = -1$ for the panels on the right. The fixed points are located at $x = 0.25$, $x = 0.5$ and $x = 0.75$. The fixation probabilities are qualitatively similar in the 2-, 3-, 4- and 5-player cases. That is, the system is more likely to fixate in one environment than the other and no novel effects due to the switching are observed. The conditional fixation times in this case are qualitatively similar to the 2-player case shown in Fig. S1.

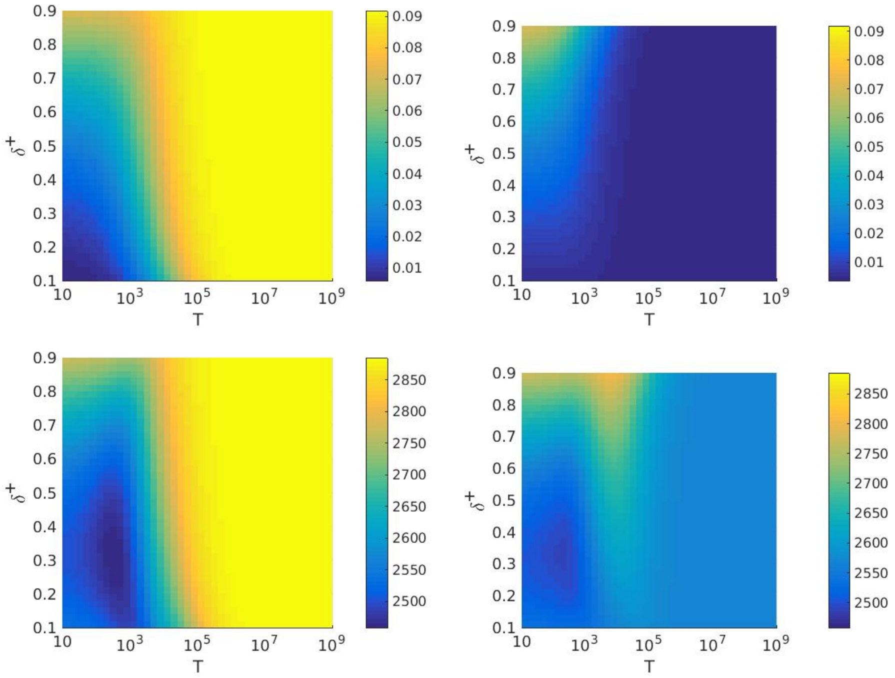

Figure S3. Fixation probabilities (top two panels) and conditional fixation times (bottom two panels) for 5-player games (4 internal fixed points). The staring environment is $\sigma = 1$ for the panels on the left and $\sigma = -1$ for the panels on the right. The fixed points are located at $x = 0.2$, $x = 0.4$, $x = 0.6$ and $x = 0.8$. The fixation probabilities are qualitatively similar in all the 2-, 3-, 4- and 5-player cases. That is, the system is more likely to fixate in one environment than the other and no novel effects due to the switching are observed. The conditional fixation times in this case are qualitatively similar to the 3-player case.

4 Environments with the same direction of flow, but different magnitudes

One may also wonder about the case where the direction of flow between the fixed points does not change between the two environments, but the strength of the flow in different regions does, as illustrated in Fig. S4.

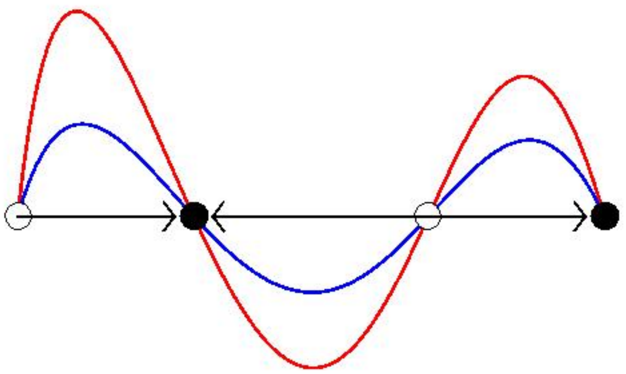

Figure S4. Strength of flow as a function of x, $\sigma = 1$ in red and $\sigma = -1$ in blue.

The fixation probabilities in Fig. S5 as a function of T and δ^+ are qualitatively similar to the the cases in Figs. S1, S2 and S3. This is to say that the $\sigma = 1$ environment is more conducive to fixation and the more time that is spent in that environment, the more likely fixation is to occur. In this case, something very similar is also true of the conditional fixation times. The mutants fixate more quickly in the $\sigma = -1$ environment than in the $\sigma = 1$ environment. This is because the stable fixed point at $x = 0.3$ is more attractive in the $\sigma = 1$ environment than in the $\sigma = -1$ environment. This can be seen from the sojourn times in Fig. S6. The interplay between the two environments here doesn't appear to produce any novel behaviour as a result of switching.

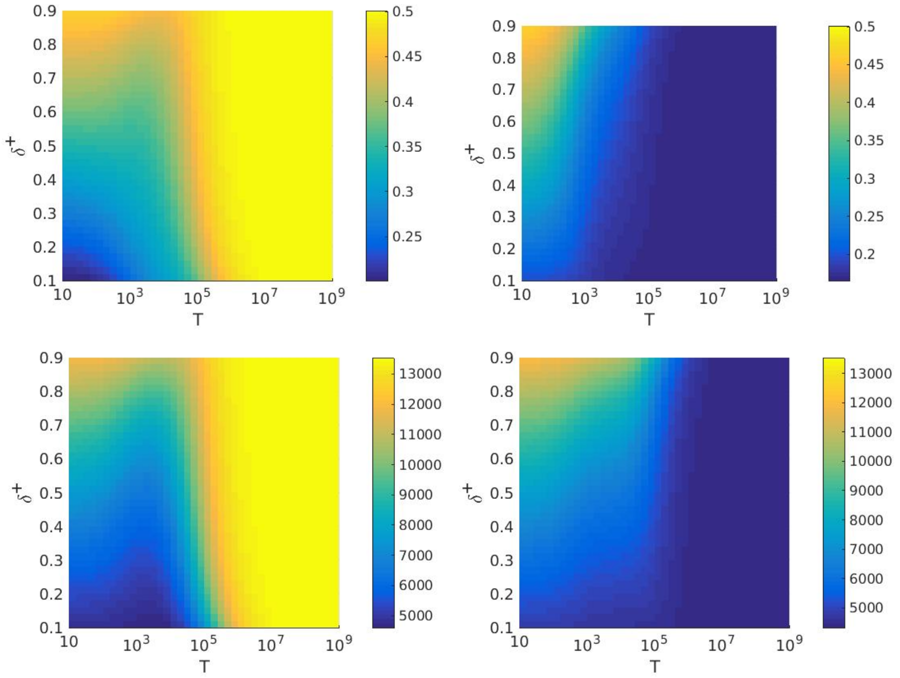

Figure S5. Results for the case where the two environments have the same arrangements of fixed points but with differing strength of flow. (Top) Fixation probability (bottom) Conditional fixation time. (Left) starting environment $\sigma = 1$, (right) starting environment $\sigma = -1$. These results indicate that the $\sigma = 1$ environment gives the higher probability of fixation whereas the $\sigma = -1$ environment gives the lower conditional fixation time. There doesn't appear to be any novel behaviour, such as global minima or maxima, that occurs as a result of switching in this case.

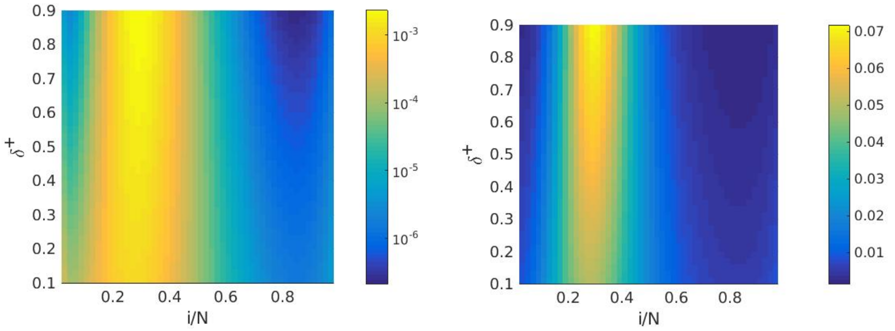

Figure S6. Unconditional sojourn times (left) and conditional sojourn times (right). As the amount of time spent in the $\sigma = 1$ environment is increased, the effective strength of the stable fixed point at $x = 0.3$ is also increased. This acts to both decrease the likelihood of absorption at the $x = 0$ boundary and increase the conditional sojourn time.
